# A genetic and clinical study of individuals with nonsyndromic retinopathy consequent upon sequence variants in *HGSNAT*, the gene associated with Sanfilippo C mucopolysaccharidosis

**DOI:** 10.1002/ajmg.c.31822

**Published:** 2020-08-07

**Authors:** Elena R. Schiff, Malena Daich Varela, Anthony G. Robson, Karen Pierpoint, Rola Ba-Abbad, Savita Nutan, Wadih M. Zein, Ehsan Ullah, Laryssa A. Huryn, Sari Tuupanen, Omar A. Mahroo, Michel Michaelides, Derek Burke, Katie Harvey, Gavin Arno, Robert B. Hufnagel, Andrew R. Webster

**Affiliations:** 1 Genetics Service, Moorfields Eye Hospital, London, UK; 2 UCL Institute of Ophthalmology, London, UK; 3 Ophthalmic Genetics and Visual Function branch, National Eye Institute, National Institutes of Health, Bethesda, Maryland; 4 Department of Electrophysiology, Moorfields Eye Hospital, London, UK; 5 North Thames Genomic Laboratory Hub, Great Ormond Street NHS Foundation Trust, London, UK; 6 Blueprint Genetics, Espoo, Finland; 7 Section of Ophthalmology, King’s College London, London, UK; 8 Enzyme Unit, Chemical Pathology, Paediatric Laboratory Medicine, Great Ormond Street Hospital for Children NHS Foundation Trust, London, UK

**Keywords:** HGSNAT, inherited retinal disease, retinopathy

## Abstract

Pathogenic variants in the gene *HGSNAT* (heparan-*α*-glucosaminide *N*-acetyltransferase) have been reported to underlie two distinct recessive conditions, depending on the specific genotype, mucopolysaccharidosis type IIIC (MPSIIIC)—a severe childhood-onset lysosomal storage disorder, and adult-onset nonsyndromic retinitis pigmentosa (RP). Here we describe the largest cohort to-date of *HGSNAT*-associated nonsyndromic RP patients, and describe their retinal phenotype, leukocyte enzymatic activity, and likely pathogenic genotypes. We identified biallelic *HGSNAT* variants in 17 individuals (15 families) as the likely cause of their RP. None showed any other symptoms of MPSIIIC. All had a mild but significant reduction of HGSNAT enzyme activity in leukocytes. The retinal condition was generally of late-onset, showing progressive degeneration of a concentric area of paramacular retina, with preservation but reduced electroretinogram responses. Symptoms, electrophysiology, and imaging suggest the rod photoreceptor to be the cell initially compromised. *HGSNAT* enzymatic testing was useful in resolving diagnostic dilemmas in compatible patients. We identified seven novel sequence variants [p.(Arg239Cys); p.(Ser296Leu); p.(Phe428Cys); p.(Gly248Ala); p.(Gly418Arg), c.1543–2A>C; c.1708delA], three of which were considered to be retina-disease-specific alleles. The most prevalent retina-disease-specific allele p.(Ala615Thr) was observed heterozygously or homozygously in 8 and 5 individuals respectively (7 and 4 families). Two siblings in one family, while identical for the *HGSNAT* locus, but discordant for retinal disease, suggest the influence of trans-acting genetic or environmental modifying factors.

## 1 | INTRODUCTION

Inherited retinal dystrophy (IRD) denotes a phenotypically and genetically heterogeneous group of disorders causing retinal dysfunction with or without retinal degeneration. They are associated with over 200 genes acting in a Mendelian fashion causing retinal disease, either uniquely or in association with systemic or syndromic disease. Collectively, IRD is the most frequent cause of blindness in the working-age population, at least in England and Wales (Liew, Michaelides, & Bunce, 2014). Retinitis pigmentosa (RP), or synonymously, rod-cone dystrophy, is in turn the most prevalent form of IRD and is due to pathology primarily and initially of rod photoreceptors. Generally, patients with RP first experience loss of night vision (nyctalopia) followed by progressive loss of peripheral vision. Central vision can be preserved in some affected individuals. The age of onset and degree of sight impairment is hugely variable.

Distinct genotypes in several autosomal genes, acting in a recessive Mendelian fashion (e.g., *USH2A*, *FLVCR1*, *CEP290*, *CLN3*, and *MFSD8*) have been shown to be associated with both syndromic or nonsyndromic forms of RP, depending on the specific component alleles. In certain genes, hypomorphic alleles have been identified that are associated with the nonsyndromic forms such as *USH2A* (Lenassi et al., 2015; Rivolta, Sweklo, Berson, & Dryja, 2000). Specific genotypes of *HGSNAT* (encoding heparan-*α*-glucosaminide *N*-acetyltransferase) in which sequence variants generally cause mucopolysaccharidosis type IIIC (MPSIIIC) or Sanfilippo C syndrome (MIM 252930)—a severe childhood onset lysosomal storage disorder—have also been associated with iso lated retinal disease in a few reported individuals.

There are four clinically indistinguishable (Valstar, Marchal, Grootenhuis, Colland, & Wijburg, 2011) subtypes of autosomal recessive MPSIII—A, B, C, and D—each caused by deficiency of a different enzyme involved in the stepwise degradation of heparan sulfate, a glucosaminoglycan (GAG). The membrane-bound HGSNAT (E.C. 2.3.1.3) catalyzes the transmembrane acetylation of the terminal glucosamine residue of heparan sulfate (Bame & Rome, 1986). Pathogenic variants in *HGSNAT* lead to the accumulation of unacetylated heparan sulfate in the lysosomes of all tissues and organs and to its excretion in the urine (Hrebícek et al., 2006).

Predominant features of MPSIIIC are progressive behavioral difficulties including hyperactivity, aggression and progressive mental deterioration leading to severe dementia. Other signs and symptoms include sleep disorders, coarse facial features, full lips, thick eyebrows, hearing impairment, vision loss (retinopathy), and seizures. The age of death for typical disease is at the end of the second or beginning of the third decade of life (Valstar, Ruijter, van Diggelen, Poorthuis, & Wijburg, 2008). In 2015, three families with specific *HGSNAT* genotypes were first associated with isolated late-onset slowly progressive retinal disease (Haer-Wigman et al., 2015) and this was followed by several reports, each of a few additional families (Carss et al., 2017; Comander et al., 2017; Long et al., 2020; Van Cauwenbergh et al., 2017).

In this study we report 20 patients from 18 families with mild or late-onset nonsyndromic RP, in whom *HGSNAT*-associated disease was explored, including clinical findings and results of enzymatic assays and genetic testing. For 17 of these patients (15 families), variants in *HGSNAT* were felt to be the cause of their disorder.

## 2 | METHODS

### 2.1 | Patients and genetic analysis

This study adhered to the tenets of the Declaration of Helsinki and was approved by the Institutional Review Boards and ethics committees of Moorfields Eye Hospital (MEH) and the National Eye Institute (NEI NCT02471287). Informed consent was obtained from all participants prior to inclusion in the study. We studied 20 patients clinically diagnosed with late onset RP: MEH1 to MEH16 attended the inherited retinal disease clinics at Moorfields Eye Hospital and underwent whole genome sequence analysis through participation in the National Institute of Health Research BioResource Rare Diseases (NIHRBR-RD) study (Carss et al., 2017) or the 100,000 Genomes Project (Turnbull et al., 2018). MEH8, a sibling of MEH7 underwent Sanger sequencing of familial *HGSNAT* variants (Manchester Centre for Genomic Medicine) and MEH16 underwent clinical exome sequencing and analysis of a panel of retinal genes (https://panelapp.genomicsengland.co.uk/panels/307/, North Thames Genomic Hub, Great Ormond Street Hospital, London). Patients NEI-1 to NEI-4 attended the National Eye Institute (NEI) and underwent sequencing of panels of 280 (Molecular Vision Laboratory) or 266 (Blueprint Genetics) retinal dystrophy genes.

### 2.2 | Ophthalmic and electrophysiological assessment

Ophthalmic examination included visual acuity (VA, using Snellen visual acuity charts), color vision (Ishihara plates) and color fundus photography, either 35° (Topcon Great Britain Ltd, Berkshire, UK) or ultra-widefield (200°) confocal scanning laser imaging (Optos plc, Dunfermline, UK). Fundus autofluorescence was performed with 30 or 55° Spectralis (Heidelberg Engineering Ltd, Heidelberg, Germany), or ultra-widefield Optos (Optos plc) imaging with excitation wavelength 488 and 532 nm, respectively. Spectral-domain optical coherence tomography (OCT) scans (Spectralis; Heidelberg Engineering Ltd) and kinetic visual fields (Goldmann or Octopus 900 Perimeter; Haag-Streit) were performed.

Electrophysiological testing included full-field and pattern electroretinography (ERG; PERG) and incorporated the International Society for Clinical Electrophysiology of Vision (ISCEV) standards (Mcculloch et al., 2015). Pattern ERG testing included recordings to standard (15 × 11°) and large (30 × 22°) stimulus fields (Lenassi, Robson, Hawlina, & Holder, 2012) Additional On–Off ERGs (Sustar et al., 2018) and S-cone ERGs (Perlman, Kondo, Chelva, Robson, & Holder, 2020) were performed according to previously described methods (Audo et al., 2008; Georgiou et al., 2019).

### 2.3 | Enzyme activity

Acetyl-CoA-D-glucosamine *N*-acetyltransferase activity (HGSNAT) was measured in leucocyte homogenates (sonicated and solubilized in triton) using the synthetic fluorimetric substrate, 4-methylumbelliferyl-β-D-glucosaminide (Moscerdam) (Voznyi et al., 1993). Protein content was assayed using bicinchoninic acid and enzyme activity was expressed in the homogenates per mg of protein. Total β-hexosaminidase was also measured in the same leucocyte homogenate as a control for sample integrity using the synthetic fluorimetric substrate, 4-methylumbelliferyl-2-acetamido-2-deoxy-β-D-gluco-pyranoside (Melford). The reference range for normal HGSNAT activity is 0.6–4.2 nmol/hr/mg protein for MEH patients. The reference range in MPSIIIC patients with typical disease and two disease causing *HGSNAT* variants is 0.013–0.02 nmol/hr/mg protein. The reference range for normal HGSNAT activity for the NEI patient is 5.8–−45 nmol/17 hr/mg protein.

### 2.4 | Quantitative determination of glycosaminoglycans in urine

Total glycosaminoglycans were quantified in random urine samples using the colorimetric dye, 1,9-dimethylmethylene blue (Sigma) using a chondroitin sulfate standard (Sigma) (de Jong, Wevers, Laarakkers, & Poorthuis, 1989). Levels were expressed as a ratio to creatinine which was measured using an enzymatic dry slide assay (Vitros, Ortho Clinical Diagnostics). The reference interval for unaffected individuals (no MPSIIIC) is 1–5 mgGAG/mmol creatinine for MEH patients for patients greater than 15 years old, and 0.6.5 mgGAG/mmol creatinine for the NEI patient.

## 3 | RESULTS

Family and patient details including clinical findings, enzymatic analysis, and *HGSNAT* alleles are summarized in Table 1 and variant details are summarized in Table 2.

### 3.1 | Clinical findings

**FIGURE 1:**
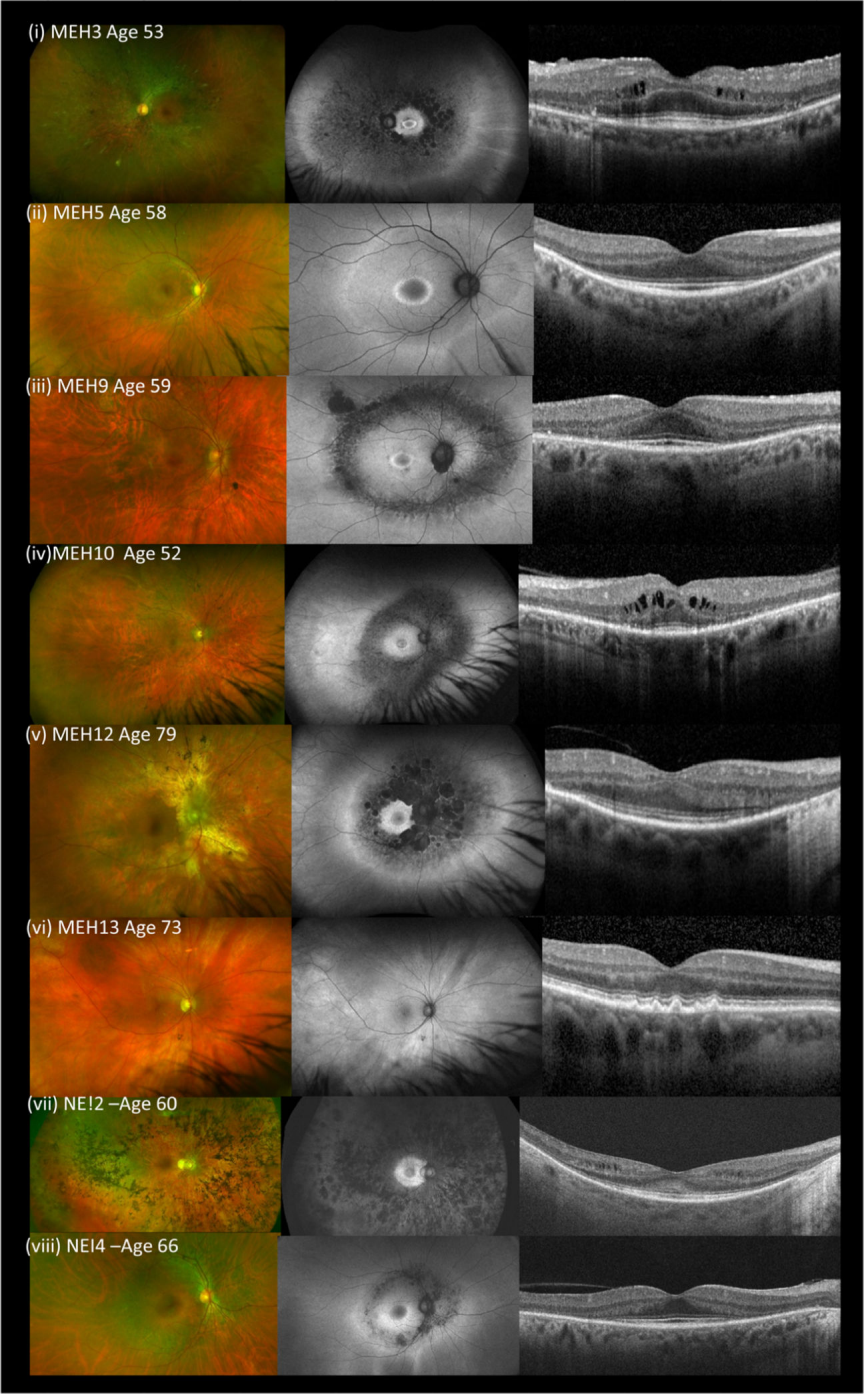
Retinal imaging—color, autofluorescence (Optos), foveal optical coherence tomography (OCT) (Heidelberg Spectralis) for patients MEH3, MEH5, MEH9, MEH10 (a)–(d) and for (e) affected (MEH12) and (f) unaffected (MEH13) siblings HOM for p.Ala615Th, and for patients NEI2 and NEI4 (g and h)

Considering the 16 affected individuals of 15 families with biallelic *HGSNAT* variants, all patients had RP with no other symptoms of MPSIIIC. Specifically, none showed any dysmorphic facial features, signs of neurological or behavioral decline or other systemic features including intellectual disability. Their retinal dystrophy was characterized in all but two cases by adult-onset, slowly progressive degeneration of the mid-peripheral retina. All had initial symptoms of nyctalopia and visual field constriction and in most cases, onset in the fourth to seventh decades of life. Visual acuity was variable with severe reduction in a number of patients while other members of the cohort retained good acuity levels. Fundus photographs, FAF imaging and OCT are shown in Figure 1. Cystoid macular edema was a feature in some. Imaging showed loss of the ellipsoid-line and outer-nuclear layer with retained autofluoresence in the outer macular in less severely affected individuals, suggesting loss of rod photoreceptors as the primary degenerative event.

**FIGURE 2:**
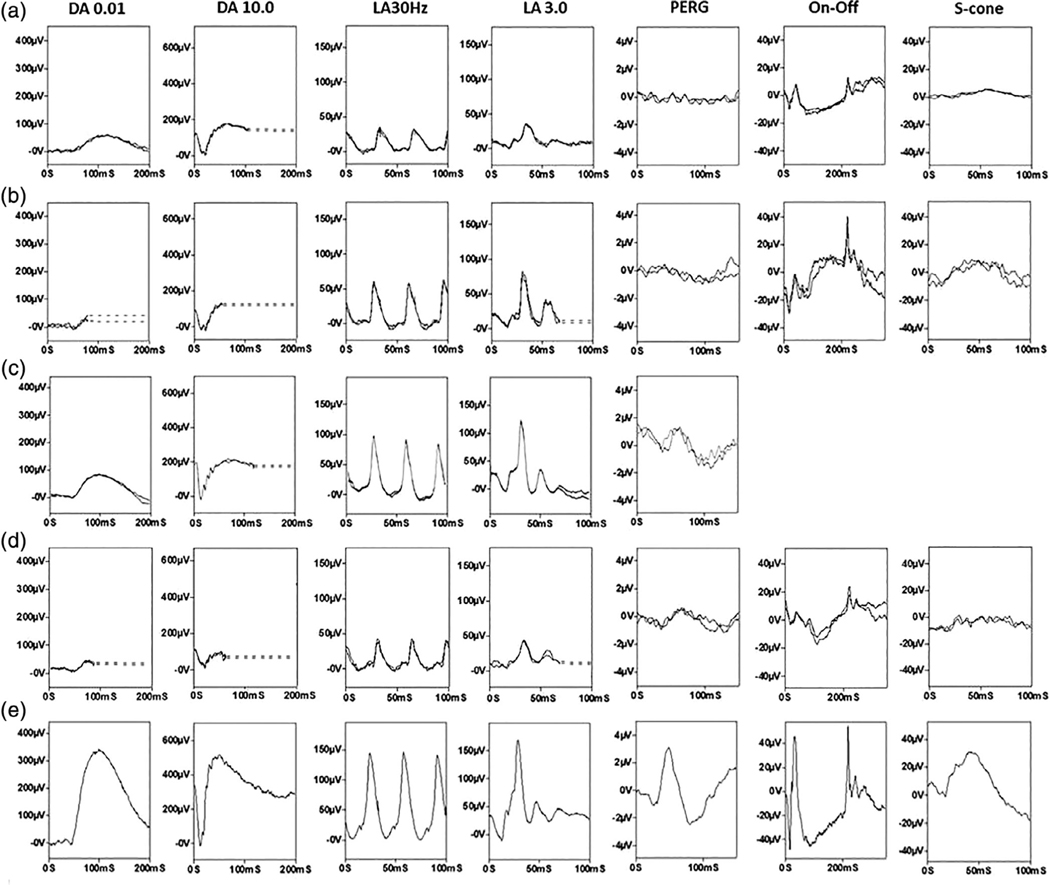
Full-field ERG and PERG findings in cases MEH1 (a), MEH4 (b), MEH5 (c) and MEH12 (d) compared with a representative unaffected control subject (e). Full-field ERGs include the dark-adapted (DA) ERGs (flash strengths 0.01 and 10.0 cd s/m^2^; DA 0.01 and DA 10.0) and light-adapted (LA) ERGs for a flash strength of 3.0 cd s/m^2^ (LA 3.0; 30 and 2 Hz). The PERG is recorded to an alternating chequerboard (15 × 11°). The full-field ERGs show evidence of similar rod and cone dysfunction (a), rod > cone dysfunction (b and d) or a loss of rod function with preserved peripheral cone system function (c). PERG P50 shows reduction, in keeping with severe (a, b) or relatively mild (c, d) macular dysfunction. There was a high degree of interocular symmetry and recordings are shown from the right eye only. Patient traces are superimposed to demonstrate reproducibility. Broken lines replace blink artifacts for clarity

Of the nine patients who had electrodiagnostic testing, full-field ERG (Figure 2), indicated generalized photoreceptor dysfunction confined to the rod system (*N* = 2); mild and similar rod and cone system involvement (*N* = 1), rod-cone dysfunction that ranged from mild to severe (*N* = 5) or undetectable responses consistent with a severe loss of rod and cone function (*N* = 1). Of the five patients who underwent standard-field PERG testing of macular function, P50 was undetectable (*N* = 2), subnormal (*N* = 3; including one with additional delay) or normal (*N* = 1), but all five had a subnormal response to a large field size, in keeping with severe paracentral macular involvement. It is highlighted that macular function could not be predicted from the severity of the full-field ERG findings; two of those with relatively mild rod and cone dysfunction had undetectable PERGs, suggesting severe macular dysfunction (Figure 2a,b).

### 3.2 | Enzymatic analysis

Leukocyte HGSNAT activity and urinary GAG/creatinine ratio were assessed in 16 of the 20 patients (Table 1). Enzyme activity was decreased in 13 of the 16 patients (0.1–0.4 nmol/hr/mg protein for MEH patients and 2.9 nmol/17 hr/mg for the NEI patient) compared with the healthy control ranges (0.64–4.2 and 5.8–45 for MEH and NEI, respectively) and increased compared with the observed range in MPSIIIC patients (0.013–0.02). MEH14, MEH15, and MEH16 had enzyme activities well within the normal range (3.3, 0.8, and 2.1 nmol/hr/mg protein, respectively), indicating that any *HGSNAT* variants were benign.

### 3.3 | **HGSNAT** variant findings in our cohort of nonsyndromic RP patients

About 20 patients (18 families) were assessed in this study, 17 of whom (15 families), were found to have biallelic *HGSNAT* variants as the most likely cause of their RP. Of the 30 alleles in these families, 12 distinct variants were observed, detailed in Table 2.

**FIGURE 3:**
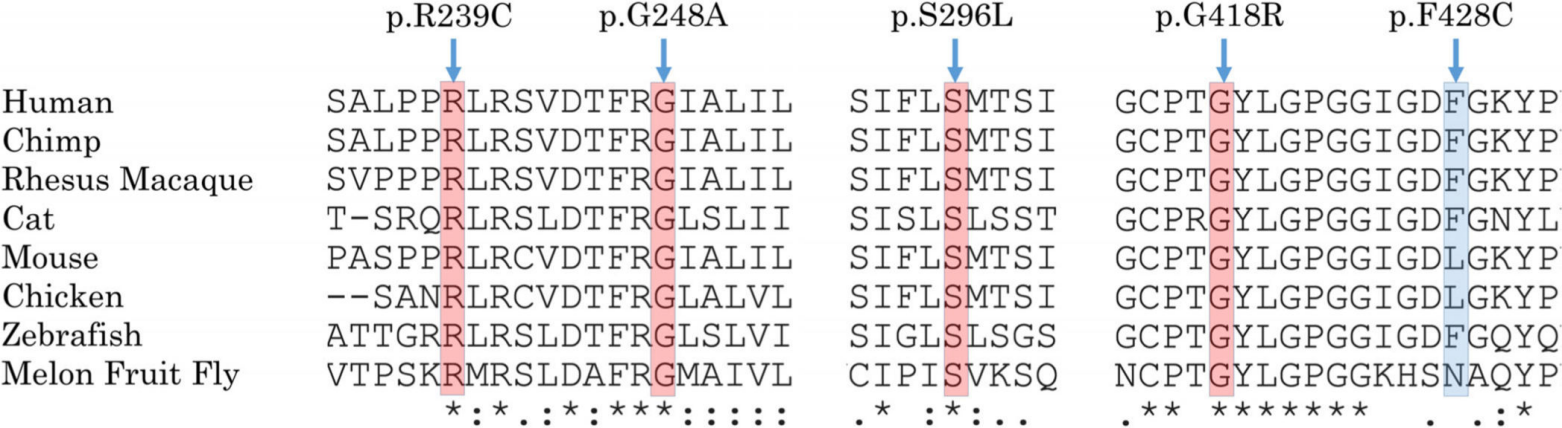
Multiple organism sequence alignment of regions spanning the R239, G248, S296, G418, and F428 amino acids of the HGSNAT protein, showing their conservation, highly conserved in red and moderately conserved in blue. Alignment was performed using https://www.uniprot.org/align/. Protein sequences used for alignment are Q68QP4–2 in the human, KZBY75 in the chimp, H9EWF5 in the Rhesus macaque, F1NBK1in the cat, M3X793 in the mouse, Q3UDW8 in the chicken, F1Q893 in the zebrafish, and A0A0A1XN23 in the Melon fruit fly

Seven of the 12 distinct variants have not previously been reported in patients (five missense variants, one splice acceptor, and one frameshift), and were found in seven patients (from seven different families). Three of the missense variants were present homozygously; p.(Ser296Leu) in MEH1, p.(Phe428Cys) in MEH2, and p. (Arg239Cys) in NEI-2. p.(Ser296Leu) was also identified as a compound heterozygote with the missense p.(Gly248Ala) in MEH3. The other three unreported variants—missense p.(Gly418Arg), splice acceptor c.1543–2A > C, and frameshift p.(Thr570ProfsTer8) were all identified as compound heterozygotes with the reported hypomorphic variant p.(Ala615Thr) in patients MEH5, MEH6, and MEH10, respectively. The peptide context of these novel missense variants in various organisms is shown in Figure 3.

About 4 of the 12 variants have been previously reported in patients with MPSIIIC as compound heterozygotes with other pathogenic missense or nonsense variants, and in this study were found in five patients (four families) in trans with the p.(Ala615Thr) variant. These variants were p.(Pro283Leu) in MEH4 (Fedele & Hopwood, 2010; Feldhammer, Durand, Mrázová, et al., 2009b; Hrebícek et al., 2006); c.1542+4dupA (Feldhammer, Durand, Mrázová, et al., 2009b) in sisters MEH7 and MEH8; c.1250+1G>A (Feldhammer, Durand, Mrázová, et al., 2009b; Fernández-Marmiesse et al., 2014; Hrebícek et al., 2006) in MEH9 and p.(Ile425HisfsTer45) (Feldhammer, Durand, Mrázová, et al., 2009b; Martins et al., 2019) in MEH11. MEH4 and MEH7 have been previously reported in a large cohort analysis of WGS (Carss et al., 2017). The frameshift p. (Ile425HisfsTer45) in MEH11, who originates from Greece, has been reported in four other MPSIIIC patients who likewise all originated from Greece (Martins et al., 2019), further supporting the common ethnic origin of this variant.

The most common variant in the cohort was p.(Ala615Thr), observed heterozygously in eight patients from seven families, and homozygously in five patients from four families (siblings MEH12 and MEH13, NEI-1, NEI-3, and NEI-4). Siblings MEH12 and MEH13 were discordant for phenotype, MEH12 had nonsyndromic mild RP with mid-peripheral field loss, with onset in his 60s and slow progression while MEH13, at age 73 was asymptomatic with a normal retinal examination (Figure 1e,f). Together with the other p.Ala615Thr homozygous patient who also underwent enzyme analysis (NEI-3), all three showed decreased HGSNAT enzyme activity and urinary GAG/creatinine ratio within the normal range, similar to the other *HGSNAT*-associated retinopathy patients in this study.

Of the three remaining unrelated patients in our cohort, two (MEH14 and MEH15) had benign *HGSNAT* variants and other causes of their RP: MEH14 who was mixed Caucasian and African-Caribbean was a compound heterozygote for two missense variants p.(Thr417Ile) and p.(Glu587Lys), reported, respectively, as benign and likely benign in ClinVar and predicted in silico to be benign (polyphen)/tolerated (SIFT) and benign/ deleterious respectively. Both p.(Thr417Ile) and p. (Glu587Lys) are similarly quite common in Africans (0.0045 and 0.0043 in gnomAD African alleles, respectively) and also both present in one unaffected brother (therefore likely to be *in-cis*, parents were not segregated) and therefore unlikely to be the cause of his RP. This was confirmed by showing HGSNAT activity within the normal range (3.3 nmol/hr/mg protein) in this patient.

MEH15 was one of the two outliers in terms of age of onset. His symptoms of difficulty in the dark and with bright light began at age 10 and by age 19 his VA was hand movements. He was homozygous for the rare (0.000003099) in gnomAD) synonymous *HGSNAT* variant of unknown significance (ClinVar), p.(Ser376=), which has a low confidence prediction of loss of function (pLoF), but he also harbored a homozygous *PROM1* stop-gain pathogenic reported variant [c.1726C>T, p.(Gln576Ter)] which instead was the likely cause of his retinal disorder. In addition, the HGSNAT enzyme activity was well within the normal range.

No *HGSNAT* variants were identified in patient MEH16. This patient’s ophthalmic findings were consistent with *HGSNAT*-associated RP, however, HGSNAT activity was well within the normal range (2.1 nmol/hr/mg protein), so while no pathogenic variants were identified using the 236-gene RETINAL panel of a clinical exome not including *HGSNAT*, it was not necessary to proceed to *HGSNAT* gene sequencing.

## 4 | DISCUSSION

In this study we describe the largest cohort to date of nonsyndromic RP associated with variants in *HGSNAT*. We identify seven novel sequence variants and four previously reported MPSIIIC variants which were in trans with the hypomorphic allele p.(Ala615Thr), thus expanding the phenotypic and genotypic spectrum of *HGSNAT-*associated retinopathy. Furthermore we identify a homozygous p. (Ala615Thr) variant in a patient and in their unaffected 73-year-old younger sibling, suggesting the influence of transacting genetic and/or environmental modifiers on the retina. We also highlight the clinical utility of simple enzymatic testing to verify the molecular diagnosis in patients with a consistent phenotype with or without rare variants in the *HGSNAT* gene.

The genotypes associated with retinal disease identified in this study support the model that the two distinct phenotypes of MPSIIIC and nonsyndromic retinal dystrophy arise due to two nonoverlapping classes of *HGSNAT* genotype. As far as the authors are aware there are no reports of identical genotypes causing both disorders. It is likely that broadly speaking those genotypes that completely, or almost completely, abolish HGSNAT function lead to the more severe early onset disorder. This is supported by biallelic null genotypes being seen in some MPSIIIC families but never in those with late-onset retinal disease. In this severe genotype class, there may be further, more subtle genotype–phenotype correlations, with frameshifts, nonsense and canonical-splice variants and missense variants giving rise to rapidly progressing forms, while some combinations of other noncanonical splicing and missense variants may give rise to slower progressing forms (Feldhammer, Durand, & Pshezhetsky, 2009a; Martins et al., 2019; Ruijter et al., 2008).

**FIGURE 4:**
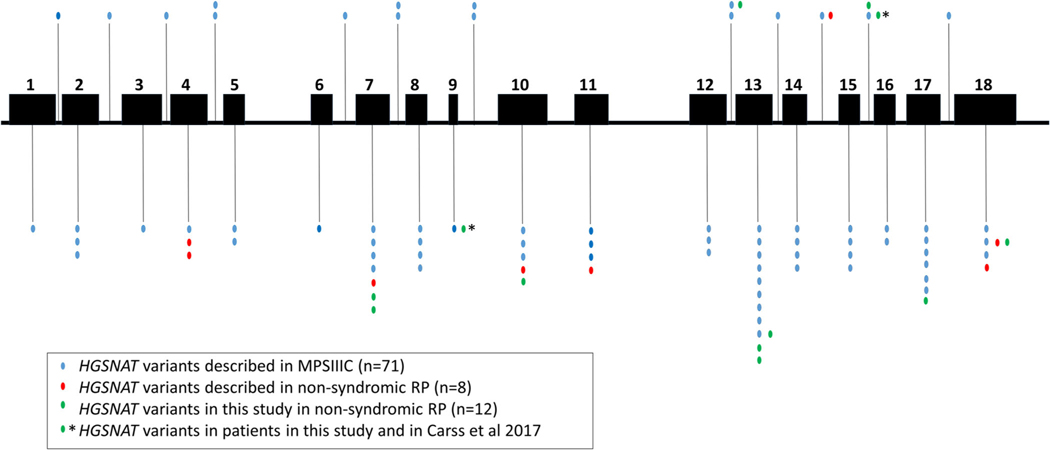
Schematic representation of the *HGSNAT* gene showing the localization and distribution of variants associated with MPSIIIC and with nonsyndromic retinitis pigmentosa (RP). Exonic variants are represented below and intronic variants above. Each vertical dot represents a unique variant. Blue dots represent the 71 variants associated with MPSIIIC described to date (Martins et al., 2019). Red dots represent the 8 reported variants associated with nonsyndromic RP in **HGSNAT** of which 6 were novel (see text). Green dots represent the 12 variants associated with nonsyndromic retinitis pigmentosa in **HGSNAT** described in this study, 7 of which are novel (see Table 2)

Observations of the genotypes in our cohort and those previously reported, suggest a class of allele that when paired together, or in trans with a null, give rise to the retina-only phenotype. This is analogous to the situation for genes such as *USH2A* (Lenassi et al., 2015; Rivolta et al., 2000) and *CLN3* (Ku et al., 2017) where specific alleles confer nonsyndromic disease when together or paired with severe alleles. The assignment to this class of allele would be made if it is seen homozygously in a retinal patient, or else in trans with a null, or previously characterized MPSIIC allele, in a retinal patient. Inspection of our data suggests that the following alleles are potentially associated with nonsyndromic retinal disease: p.(Ala615Thr), p.(Arg239Cys), p.(Ser296Leu), and p.(Phe428Cys). Applying these same rules to previously published genotypes, in retinal degeneration patients, would suggest that p.(Arg124Trp) identified homozygously in two families (Haer-Wigman et al., 2015), and p.(Ser318Asn), homozygous in one family (Comander et al., 2017), also belong to this class of alleles. In addition, the extension allele at the end of exon 18 in trans with a null, described recently in a Chinese family (Long et al., 2020) would also fit into this class of retinal disease-specific allele. Alleles found uniquely in retinal dystrophy patients, but only paired with another known allele of this class, could belong to either class and are here termed “undefined.” This includes both p.(Gly418Arg) and p. (Gly248Ala) in this study. The peptide position of these alleles is shown in Figure 4, in the context of all those previously reported in families with MPSIIIC (Martins et al., 2019) and retinal dystrophy (Table S1). There is no obvious clustering of those missense variants associated with nonsyndromic retinal disease.

The variant, p.(Ala615Thr) is of particular interest for two reasons. Firstly, it is by far, the most common *HGSNAT* allele seen in retinal degeneration patients in this study. Furthermore, data from Blueprint genetics (verbal communication ST), in which NGS panel analysis has been performed on 5,753 referred cases of retinal dystrophy shows this allele is present in 36 patients in a genotype likely to explain their molecular diagnosis (0.63%). About 14 of these were homozygous and 12 heterozygous paired with a second likely pathogenic variant in *HGSNAT*. Secondly the variant has a relatively high prevalence in the general population (gnomAD v2.1.1 accessed June 2020—minor allele prevalence = 0.00403, including four homozygotes). One explanation for this would be that such genotypes may, in some individuals, be nonpenetrant which would be relevant for genetic counseling.

In this study, we show evidence for this conjecture in two siblings—one with late onset mild RP and the other who was asymptomatic at age 73—both homozygous for p.(Ala615Thr). Further, inspection of the whole-genome data suggests both siblings are genotypically identical at the *HGSNAT* locus (no discordance for SNPs within 500 kb flanking the variant). This excludes the effect of differing haplotypic backgrounds (cis-modifiers) and instead suggests the action of, as yet unknown, trans-acting genetic or environmental modifiers. Their identification might provide clues to effective ameliorating measures or drug treatments. Further, both siblings were shown to have similarly reduced leukocyte HGSNAT enzymatic activity, suggesting these factors might be acting through photoreceptor protection, rather than affecting the underlying lysosomal pathway.

This study has demonstrated the utility of enzyme testing in supporting the molecular diagnosis, and that enzymatic activity levels appear to play an important role in determining the phenotype. Three patients MEH14, MEH15, and MEH16 had a phenotype consistent with *HGSNAT*-retinopathy, with slowly progressive degeneration of the midperipheral retina, with two having bi-allelic *HGSNAT* variants of unknown significance (MEH16 was not tested for *HGSNAT* variants). The finding that enzyme activities of all three within the normal range, suggested the variants to be benign and the retinopathies were unlikely to be related to *HGSNAT*. Regarding the threshold level for retinal disease, given the lack of reports of retinal degeneration in parents of MPSIIIC affected children, that required for nonsyndromic retinal disease might be expected to lie between 10 and 40% of normal activity.

The phenotype presented here and by others appears distinct—with a late-onset of presentation and diagnosis, a symmetrical (both inter- and intraocular) distribution of degeneration that is pericentral. The functional phenotype was more variable with full-field ERGs, ranging from undetectable to showing relatively mild loss of rod function (see Fig ERG1 A and D; both aged 70 years with relatively mild ERG abnormalities). The PERG indicated spared or relatively spared central macular function in the majority, consistent with a previous report that labeled the condition “pericentral RP” (Comander et al., 2017), but there were also exceptions, with severe macular dysfunction occurring in some with ERG evidence of mild peripheral retinal dysfunction. Depending upon the resources available, enzymology, rather than nucleotide sequencing, might be the most efficient test to secure a molecular diagnosis. The distinct phenotype also helps clinicians interpret variants found in panel, exome-, or genome-wide testing.

In conclusion, we have here expanded the phenotypic and genotypic spectrum of nonsyndromic *HGSNAT*-retinopathy, and added at least three further alleles to those that appear to confer disease that affects only the retina. The discordant siblings in our cohort suggest the action of as yet unknown trans-acting genetic and/or environmental modifiers that might determine nonpenetrance and help explain the high population prevalence of the most prevalent retinal disease specific variant.

## Supplementary Material

Table S1 HGSNAT-associated nonsyndromic retinitis pigmentosa variants in the literature

## Figures and Tables

**TABLE 1:** Clinical characteristics, enzymatic analysis, and genotype of patients with biallelic HGSNAT variants

ID family (sex) origin	Initial symptoms and age of onset (years)	BCVA (age)	Visual field	Fundus features and OCT	Macular edema	Retinal function according to ERG; standard and large field PERG; (age)	*HGSNAT* (allele 1, allele 2)	HGSNAT nmol/hr/mg ptn *(N* = 0.64–4.2: MPSIIIC = 0.013–0.02)	Urine mgGAG/mmol creatinine ratio *(N =* 1–5)
MEH121.459 (M) Pakistan	Vision deterioration; nyctalopia; visual field (62)	6/60 OD 6/60 OS (77)	Pericentral scotomata with central involvement; intact far periphery	Retinal degeneration affecting outer macular and posterior pole; preserved fovea	No	Mild rod and cone dysfunction; severe widespread macular dysfunction; (70)	c.887C>T p.Ser296Leu (homozygous)	0.4	4
MEH216.348 (F) Pakistan	Nyctalopia (55)	HM OD 1/60 OS (81)	Profound loss of central and peripheral fields	Marked central and peripheral RPE atrophy	No		C.1283T >G p.Phe428Cys (homozygous)	0.3	2
MEH321.265 (M) Punjab	Nyctalopia (30s)	6/9 OD 6/9 OS (54)	Mid-peripheral field loss	Foveal structure is normal	Yes		c.887C>T p.Ser296Leu c.743G>C p.Gly248Ala	0.1	3
MEH4^a^20,436 (F)UK	Nyctalopia (30s)	6/18 OD 6/24 OS (66)	Mid-peripheral field loss	Some atrophy adjacent to disc	Yes	Rod-cone dysfunction; severe widespread macular dysfunction; (54)	c.1843G>A p.Ala615Thr c.848C>T p.Pro283Leu	0.19	3
MEH520.741 (M)UK	Paracentral field defects (38)	6/9 OD 6/6 OS (50)	Mid-peripheral field loss	Rings of hyperautofluorescence. Retinal dystrophy involves small area in outer macular	No	Mild rod dysfunction; central macular dysfunction with severe paracentral macular involvement (43)	c.1843G>A p.Ala615Thr c.1252G>C p.Gly418Arg	0.3	2
MEH620.918 (M)UK	Nyctalopia (56)	HM OD HM OS (82)	Profound loss of peripheral and mid-peripheral fields	Healthy foveal structure	No		c.1843G>A p.Ala615Thr c.1543–2A>C	0.1	3
MEH7^b^19,963 (F)UK	Nyctalopia (47)	6/24 OD 6/6 OS (57)	Mid-peripheral field loss	Symmetric retinal degeneration in outer macular	Yes	Mild rod dysfunction; normal central macular function with severe paracentral macular involvement (49)	c.1843G>A p.Ala615Thr c.l542+4dupA	NA	2
MEH819.963 (F)UK	Nyctalopia (30s)	6/9 OD 6/12 OS (57)	Mid-peripheral field loss	Midperipheral degeneration	Yes		c.1843G>A p.Ala615Thr c.l542+4dupA	0.1	NA
MEH921.345 (M)UK	Nyctalopia; (20s)	6/5 OD 6/9 OS (59)	Mid-peripheral field loss	Pericentral RP. Retinopathy affecting midperipheral retina; foveal-spa ring	No		c.1843G>A p.Ala615Thr c.1250+1G>A	0.27	2
MEH1019.848 (M)UK	Visual field constriction; flashing lights (30s)	6/9 OD 6/6 OS (53)	Mid-peripheral field loss	Bone spiculation; retinal degeneration affecting ring of tissue around posterior pole; healthy foveal structure	Yes	NA	c.1843G>A p.Ala615Thr c.l708delA p.Thr570ProfsTer8	0.3	2
MEH1119,482 (F) Greece	Nyctalopia; visual field constriction (40s)	6/19 OD 6/24 OS (70)	Peripheral field loss	Bone spicule pigmentation	Yes		c.1843G>A p.Ala615Thr c.l271dupG p.lle425HisfsTer45	0.2	3
MEH1219.609 (M)UK	Nyctalopia; visual field constriction; ring scotoma (60s)	6/12 OD 6/9 OS (80)	Mid-peripheral field loss	Mild RP	No	Rod-cone dysfunction; macular dysfunction with severe paracentral macular involvement; (71)	c.1843G>A p.Ala615Thr homozygous	0.3	2
MEH1319.609 (F)UK	None	6/9 OD 6/6 OS (73)	Normal	Small insignificant drusen	No	NA	c.1843G>A p.Ala615Thr homozygous	0.4	2
M EH1425,243 (M) Caribbean/ White	Visual field constriction (23)	6/9 OD 6/60 OS (58)	Profound loss of peripheral and mid-peripheral fields	Widespread retinal pigmentation, attenuated vessels	Yes	NA	c.1250C>T p.Thr417 c.1759G>A p.Glu587Lys	3.3	2
MEH1523.608 (F) Pakistan	Difficulty in the dark and with bright light (10)	HM OD HM OS (19)		Retinal degeneration affecting posterior pole	No	NA	c.H28G>p. Ser376 = also homozygous for PROM1 pathogenic c.1726C>T p.Gln576Ter	0.8	2
M EH1627,085 (M)UK	Visual field defects (50)	6/6 OD 6/6 OS (52)	Full peripheral fields	Midperipheral retinal dystrophy, intact foveal structure	No	NA	NA	2.1	NA
NEI 1A(F) Ashkenazi	Nyctalopia (50)	6/9.5 OD 6/6 OS (84)	Profound loss of peripheral and mid-peripheral fields	Midperipheral bone spiculation and hy poa u tof 1 uo resce n ce. Subfoveal island of outer layers, retinal thinning	No	Severe rod-cone dysfunction; severe macular dysfunction (78)	c.1843G>A p.Ala615Thr homozygous	NA	NA
NEI 2B (M) Caucasian	Nyctalopia; visual field constriction (16)	6/60 OD 6/60 OS (60)	Profound loss of peripheral and mid-peripheral fields	Mid peripheral and peripheral bone spiculation, widespread hy poa u tof 1 uo resce n ce with central macular hyperautofluorescence. Intact foveal structure	Yes	Severe loss of rod and cone function indicated by undetectable responses; (60)	c.715C>T p.Arg239Cys homozygous	NA	NA
NEI 3C (F) North European	Scotomas (36)	6/9.5 OD 6/12 OS (46)	Profound loss of peripheral and mid-peripheral fields	Mid peripheral bone spiculation and hy poa u tof 1 uo resce n ce, mild central macular hyperautofluorescence, intact foveal structure	Yes	Moderate rod-cone dysfunction (46)	c.1843G>A p.Ala615Thr homozygous	2.91 nmol/17 hr/mg (normal 5.8–45)	4.19 mg/mmol (normal 0–6.5)
NEI 4D (M) Caucasian	None, diagnosed on routine evaluation (66)	6/6 OD 6/6 OS (66)	Mild peripheral constriction	Midperipheral retinal atrophy with few bone spicules, hy poa u tof 1 uo resce n t midperipheral ring, intact fovea 1 structure	No	Moderate-severe rod-cone dysfunction (66)	c.1843G>A p.Ala615Thr homozygous	NA	NA

Abbreviations: BCVA, Best corrected visual acuity; ERG, electroretinogram; F, female; M, male; NA, not available; OD, right eye; OS, left eye; OCT, optical coherence tomography; PERG, pattern electroretinogram.

a Patient previously reported in Carss et al., 2017, Table S2 (ID G006294).

b Patient previously reported in Carss et al., 2017, Table S2 (ID W000176).

Patients MEH7 and MEH8 are siblings; Patients MEH12 and MEH13 are siblings

**TABLE 2:** HGSNAT variants in cohort

Nucleotide	Protein	Exon	Effect	gnomAD	ClinVar	Pathogenicity	Reported
**c.715C>T**	**p.(Arg239Cys)**	7	Polyphen probably damaging, SIFT deleterious. Hydrophilic highly conserved arginine to hydrophobic cysteine just on the cytoplasmic side of the second transmembrane domain	Absent	*SCV001426183	Novel likely hypomorphic missense allele, homozygously causes RP	Novel
c743G>C	p.(Gly248Ala)	7	Splice site impact. Polyphen prob damaging, SIFT deleterious. Polar to non-polar	0.000037 in S Asians, 0 in remaining populations. Overall 0.0000087	*SCV001426184	Novel missense allele, causes RP when in trans with hypomorphic missense	Novel
**c.848C>T**	**p.(Pro283Leu)**	9	Polyphen prob damaging, SIFT deleterious. Hydrophobic to hydrophobic in membrane	0.00003234 (9)	#1232	Likely pathogenic in MPS IIIC. Causes RP when in trans with hypomorphic allele	Previously reported in MPSIIIC [as p.(Pro311Leu)] in heterozygous and homozygous state (Hrebícek et al., 2006). Functional studies show p.(Pro283Leu) has reduced enzyme activity and mislocalization due to incorrect protein folding (Fedele & Hopwood, 2010; Feldhammer, Durand, & Pshezhetsky, 2009a)
**c.887C>T**	**p.(Ser296Leu)**	10	Polyphen prob damaging, SIFT deleterious. Polar hydrophilic to non-polar hydrophobic just on edge of membrane and cytoplasm	0.0001; 32/33 in S Asians (0.001)	*SCV001426185	Novel likely hypomorphic missense allele, homozygously causes RP	Novel c.887C>A, p.(Ser296Ter) described in MPSIIIC Feldhammer, Durand, & Pshezhetsky, 2009a, origin Pakistan
c.1250 +1G>A		Intron 12	Splice site variant	0.000007 (2)	#96500	Likely pathogenic /pathogenic in MPSIIIC. Causes RP when in trans with hypomorphic allele	Reported in MPSIIIC patients with c.1270G>A, p. (Gly424Ser) (Fernández-Marmiesse et al., 2014), in Feldhammer, Durand, & Pshezhetsky, 2009a, and as c.1334+1G>A, p.(Gly446Ter) with p.(Arg412XTer) (Hrebícek et al., 2006)
**c.1252G>C**	**p.(Gly418Arg)**	13	Non-polar hydrophobic to strongly basic just inside lysosomal lumen	Absent	*SCV001426186	Novel missense allele, causes RP when in trans with hypomorphic missense	Novel
c.1271dupG	p.(Ile425HisfsTer45)	13	Frameshift in lysosomal lumen	Absent	*SCV001426187	Pathogenic in MPSIIIC. Causes RP when in trans with hypomorphic allele	Reported in two MPSIIIC patients from Greece in combination with missense mutations p.(Ser541Leu) and p.(Glu471Lys), respectively (Feldhammer, Durand, & Pshezhetsky, 2009a). Also homozygous in two Turkish siblings (Martins et al., 2019).
**c.1283T>G**	**p.(Phe428Cys)**	13	Polyphen possibly damaging; SIFT-tolerated. Nonpolar hydrophobic to polar in lysosomal lumen	0.0004 (12) in S Asians, absent in other populations in gnomAD (overall 0.000049)	*SCV001426188	Novel likely hypomorphic missense allele, homozygously causes RP	Novel
c.1542+4dupA		Intron 15	Splice site mutation, loss of function	0.000018 (5 in NFE)	#438150	Likely pathogenic in MPSIIIC. Causes RP when in trans with hypomorphic allele	Reported in MPSIIIC patient (Feldhammer, Durand, & Pshezhetsky, 2009a) in trans with nonsense mutation p. (Tyr558Ter)
**c.1543–2A>C**		Intron 15	Intron 15/exon 16 splice acceptor variant, novel	Absent	*SCV001426189	Novel splice variant causes RP when in trans with hypomorphic missense	Novel
**c.1708delA**	**p.(Thr570ProfsTer8)**	17	Frameshift in membrane	Absent	*SCV001426190	Novel frameshift causes RP when in trans with hypomorphic missense	Novel
c.1843G>A	p.(Ala615Thr)	18	Polyphen benign; SIFT deleterious. Nonpolar hydrophobic to polar hydrophilic inside membrane	0.00403 (1,119 HETs, 4 homozygotes)	#208816	Hypomorphic allele, seen in trans with previously reported MPS IIIC / novel missense/ likely pathogenic/ null alleles or homozygously	Reported (Comander et al., 2017; Fedele & Hopwood, 2010; Feldhammer, Durand, & Pshezhetsky, 2009a; Haer-Wigman et al., 2015; Hrebícek et al., 2006; Van Cauwenbergh et al., 2017)
c.1250C>T	p.(Thr417Ile)	13	Polyphen benign, SIFT tolerated. Polar hydrophilic to non-polar hydrophobic	0.0045 (110) in Africans	#719495	Benign	No citations
c.1759G>A	p.(Glu587Lys)	18	Polyphen benign, SIFT deleterious. Acidic to basic, both hydrophilic	0.0043 (101) in Africans	#719496	Likely benign	No citations
c.1128G>A	p.(Ser376=)	11	Synonymous splice region variant; low confidence pLoF	0.00002 (5)	#363146	VUS	No citations

Abbreviations: gnomAD, genome aggregation database; MPSIIIC, mucopolysaccharidosis Type IIIC; pLoF, predicted loss of function; RP, retinitis pigmentosa; VUS, variant of uncertain significance.

* Novel HGSNAT variants identified in the patients, also indicated in bold.

Reference sequence for HGSNAT is NM_152419.3, with 18 coding exons, transcript length 5,227 bps, translation length 635 amino acid residues, uniport identifier Q68CP4.
