# Supplementary material for: A genetic and clinical study of individuals with nonsyndromic retinopathy consequent upon sequence variants in HGSNAT, the gene associated with Sanfilippo C mucopolysaccharidosis
Source: Am J Med Genet C Semin Med Genet. 2020 Aug 7;184(3):631–43. doi: 10.1002/ajmg.c.31822 (PMC8125330; doi:10.1002/ajmg.c.31822)
Supplement: Supplementary file 1 — Table S1 HGSNAT‐associated nonsyndromic retinitis pigmentosa variants in the literature [file AJMG-184-631-s001.docx]

**Supplementary Table 1. HGSNAT-associated non-syndromic retinitis pigmentosa variants in the literature**

| **Reference** | **Family** | **ID** | **Age of diagnosis** | **Clinical** | **Mutation 1** | **Protein 1** | **Exon** | **Mutation 2** | **Protein 2** | **Exon** | **Enzyme activity** | **Urinary GAGs** |
| --- | --- | --- | --- | --- | --- | --- | --- | --- | --- | --- | --- | --- |
| Berger-Plantinga et al 2004 | 1 |  | 42 | RP + AO dementia | NA | NA |  | NA | NA |  | 4.1 N=13-46 | ↑ |
|  | 1 |  | 46 | RP + AO dementia | NA | NA |  | NA | NA |  | 1.4 N=13-46 | ↑ |
| Haer-Wigman et al 2015 | 2 | C-II-1 | 52 | RP + AO dementia | c.1843G>A | p.Ala615Thr | 18 | c.1843G>A in cis with c.398G>C | p.Ala615Thr in cis with p.Gly133Ala | 4 | ^a^3.6 | NA |
|  | 2 | C-II-2 | 47 | non-syndromic RP | c.1843G>A | p.Ala615Thr | 18 | c.1843G>A in cis with c.398G>C | p.Ala615Thr in cis with p.Gly133Ala | 4 | ^a^4.6 | NA |
|  | 2 | C-II-3 | 50 | non-syndromic RP | c.1843G>A | p.Ala615Thr | 18 | c.1843G>A in cis with c.398G>C | p.Ala615Thr in cis with p.Gly133Ala | 4 | ^a^3.3 | NA |
|  | 3 | A-II-1 | 34 | non-syndromic RP | c.370A>T | p.Arg124Trp | 4 | c.370A>T | p.Arg124Trp | 4 | ^a^2.5 | ^c^6.6mg/mmolcreatinine |
|  | 4 | B-II-1 | 29 |  | c.370A>T | p.Arg124Trp | 4 | c.370A>T | p.Arg124Trp | 4 | NA | NA |
|  | 4 | B-II-2 | 30 |  | c.370A>T | p.Arg124Trp | 4 | c.370A>T | p.Arg124Trp | 4 | NA | NA |
| Van Cauwenbergh et al 2017 | 5 | P33 | 47 | non-syndromic RP | c.1843G>A | p.Ala615Thr | 18 | c.634-408_820+338delinsAGAATATG | p.Glu212GlyfsTer2 | 7 | NA | NA |
| Comander et al 2017 | 6 | 15 | 55 | Pericentral RP | c.1843G>A | p.Ala615Thr | 18 | c.1464+1G>A |  | intr 14 | NA | NA |
|  | 7 | 34 | 63 | Pericentral RP | c.953G>A | p.Ser318Asn | 10 | c.953G>A | p.Ser318Asn | 10 | NA | NA |
|  | 8 | 13 | 35 | Pericentral RP | c.1843G>A | p.Ala615Thr | 18 | c.1843G>A | p.Ala615Thr | 18 | NA |  |
|  | 9 | 24 | 55 | Pericentral RP | c.1843G>A | p.Ala615Thr | 18 | c.1843G>A | p.Ala615Thr | 18 | NA |  |
| Carss et al 2017* | 10 | G006294 | 30s | non-syndromic RP | c.1843G>A | p.Ala615Thr | 18 | c.848C>T | p.Pro283Leu | 9 | NA | NA |
|  | 11 | W000176 | 47 | non-syndromic RP | c.1843G>A | p.Ala615Thr | 18 | c.1542+4dupA |  | intr 15 | NA |  |
| Long et al 2020 | 12 | II:1 | 27 | non-syndromic RP | c.1048C>T | p.Gln350* | 11 | c.1908A>G | p.*636Trpext*12 | 18 | NA |  |
|  | 12 | II:2 | 26 | non-syndromic RP | c.1048C>T | p.Gln350* | 11 | c.1908A>G | p.*636Trpext*12 | 18 | ^b^57.45nmol/h/mg protein | ^d^7.63 |
|  | 12 | II:3 | 28 | non-syndromic RP | c.1048C>T | p.Gln350* | 11 | c.1908A>G | p.*636Trpext*12 | 18 | NA |  |

*These two patients are also part of the current cohort: Shaded area represent likely hypomorphic variants (see text).

^a^ Control range 6.7-23.5 nmol/18h/mg; MPSIIIC range 0.7-3.0 nmol/18h/mg protein: ^b^33.6% healthy control levels: ^c^Control reference <3mg/mmol creatinine: ^d^Control reference<0.5mg/mmol creatine
